# Revolutionizing Drug Targeting Strategies: Integrating Artificial Intelligence and Structure-Based Methods in PROTAC Development

**DOI:** 10.3390/ph16121649

**Published:** 2023-11-24

**Authors:** Danishuddin, Mohammad Sarwar Jamal, Kyoung-Seob Song, Keun-Woo Lee, Jong-Joo Kim, Yeong-Min Park

**Affiliations:** 1 Department of Biotechnology, Yeungnam University, Gyeongsan 38541, Republic of Korea; danish23@yu.ac.kr; 2 BioWiz Laboratories, Inc., Ste-466440 Burroughs St., Detroit, MI 48202, USA; sarwar4u@gmail.com; 3 Department of Medical Science, Kosin University College of Medicine, 194 Wachi-ro, Yeongdo-gu, Busan 49104, Republic of Korea; kssong@kosin.ac.kr; 4 Division of Life Science, Department of Bio & Medical Big-Data (BK4 Program), Research Institute of Natural Science (RINS), Gyeongsang National University (GNU), 501 Jinju-daero, Jinju 52828, Republic of Korea; 5 Angel i-Drug Design (AiDD), 33-3 Jinyangho-ro 44, Jinju 52650, Republic of Korea; 6 Department of Integrative Biological Sciences and Industry, Sejong University, 209, Neugdong-ro, Gwangjin-gu, Seoul 05006, Republic of Korea

**Keywords:** PROTACs, E3 ligases, linker, artificial intelligence

## Abstract

PROteolysis TArgeting Chimera (PROTAC) is an emerging technology in chemical biology and drug discovery. This technique facilitates the complete removal of the target proteins that are “undruggable” or challenging to target through chemical molecules via the Ubiquitin–Proteasome System (UPS). PROTACs have been widely explored and outperformed not only in cancer but also in other diseases. During the past few decades, several academic institutes and pharma companies have poured more efforts into PROTAC-related technologies, setting the stage for several major degrader trial readouts in clinical phases. Despite their promising results, the formation of robust ternary orientation, off-target activity, poor permeability, and binding affinity are some of the limitations that hinder their development. Recent advancements in computational technologies have facilitated progress in the development of PROTACs. Researchers have been able to utilize these technologies to explore a wider range of E3 ligases and optimize linkers, thereby gaining a better understanding of the effectiveness and safety of PROTACs in clinical settings. In this review, we briefly explore the computational strategies reported to date for the formation of PROTAC components and discuss the key challenges and opportunities for further research in this area.

## 1. Introduction

**Figure 1:**
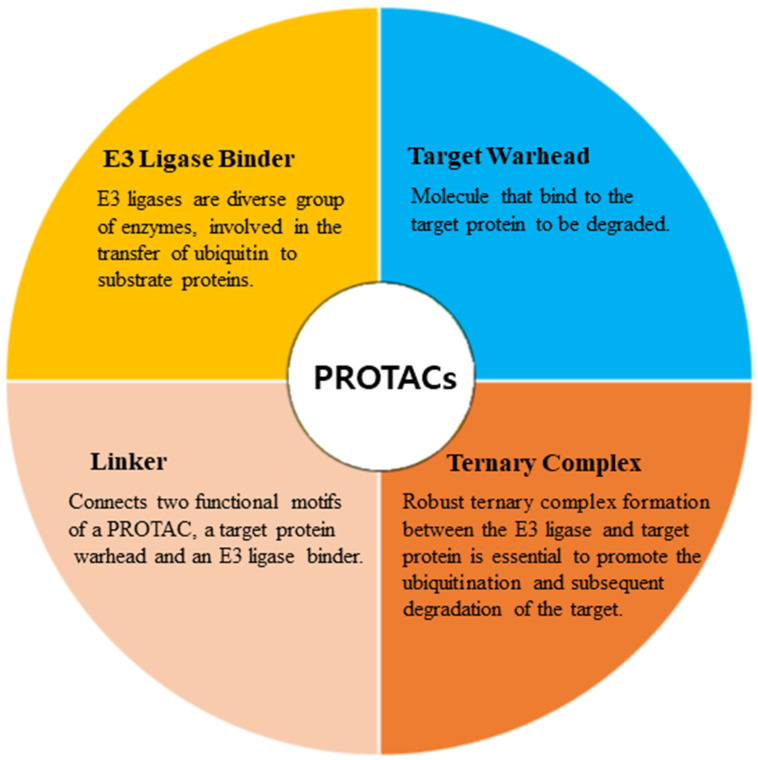
Major components involved in the design of PROTACs.

**Figure 2:**
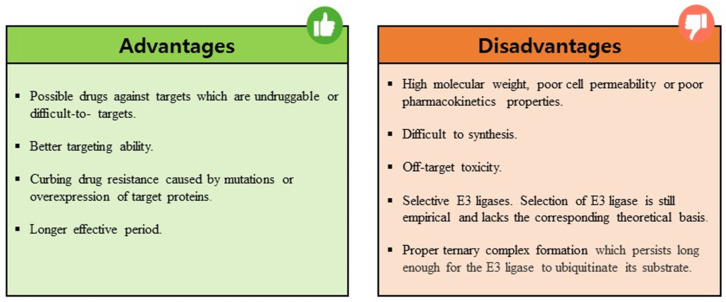
Summary of the advantages and disadvantages associated with using PROTACs as a therapeutic strategy.

PROTACs are emerging as a promising therapeutic strategy to treat different diseases, including cancer, neurological disorders, and several viral infections [1]. The approach entails utilizing small molecules that can selectively induce the degradation of specific target proteins, thereby providing a potentially effective treatment option [1,2]. These molecules work by inducing the degradation of targeted disease proteins by harnessing the cell’s proteasomal machinery [3]. The proposed strategy involves the utilization of two distinct domains within a molecule. One domain is specifically designed to bind to the target protein of interest (POI), while the other domain is responsible for binding to an E3 ubiquitin ligase enzyme. The E3 ligase is crucial for initiating the degradation of the target protein through the 26S proteasome system. This two-domain structure of PROTACs allows for targeted protein degradation and holds significant potential for therapeutic applications (Figure 1). A linker serves as the bridge between the two domains within a PROTAC molecule, facilitating the formation of a ternary complex involving the target protein and the E3 ligase [4,5]. PROTACs offer numerous advantages compared to conventional small molecule inhibitors (Figure 2). One notable advantage is their capability to target protein–protein interactions, allowing for the disruption of specific protein complexes involved in disease processes [6]. Furthermore, they also exhibit selectivity in inducing the degradation of disease-associated proteins while sparing normal cellular proteins. This selective action enhances their therapeutic potential by reducing side effects that may arise from non-specific protein inhibition [7]. Moreover, PROTACs can overcome drug resistance by targeting multiple sites on the target protein, thereby reducing the likelihood of mutations that can confer resistance [7,8].

## 2. PROTAC Advancement

The development of PROTACs has been made possible by recent advances in chemical biology and proteomics, which have allowed for the identification of specific E3 ligases that can be harnessed for the degradation of disease-causing proteins [9,10]. PROTACs have made significant advancements over the last two decades in the targeted degradation of various proteins [11,12,13]. Currently, several PROTACs are approaching either phase 1 or phase 2 clinical trials [12]. The Protein Data Bank (https://www.rcsb.org/) is experiencing an exponential increase in the number of available PROTAC ternary structures. This expansion provides a valuable resource for the development of knowledge-based strategies to enhance the success rates of ternary complex formation and optimize PROTAC design. Furthermore, the analysis of these crystal structures yields significant insights into the potential interactions among the E3 ligase, target protein, and components of PROTAC. In recent years, dedicated databases for PROTACs have also emerged. One such resource is PROTACpedia (https://protacpedia.weizmann.ac.il/ptcb/main, accessed on 17 October 2023), which provides free access to manually curated data on 1189 PROTACs, including information on their linker and activity class (active or inactive). PROTAC-DB [14] is a comprehensive database that provides extensive information on PROTACs, including their chemical structures, biological activities, and physicochemical properties. This valuable resource offers detailed insights into the characteristics and properties of these molecules, facilitating research and development in the field of targeted protein degradation. The latest release of PROTAC-DB by Weng and colleagues [15] is a significant upgrade from the previous version. The database now contains 3270 PROTACs (as accessed on 16 October 2023), representing a 96% increase in the number of compounds. Moreover, the database includes 365 warheads, 1501 linkers, 82 E3 compounds, and 664 ternary models, providing a wider selection of small molecules for protein degradation studies. The PROTAC–Model method is also introduced to predict the ternary complex structures for PROTACs, which is valuable given the limited availability of crystal target-PROTAC-E3 ternary complex structures. In addition, a new filtering strategy based on E3 ligases has been added to facilitate PROTAC data analysis.

## 3. PROTAC Advantages

The main advantage of PROTAC technology is that it enables the development of drugs for challenging and undruggable targets. Unlike traditional inhibitors, PROTACs degrade the target rather than binding to its active site or specific protein interactions. They do not require a strong affinity for the target, thus reducing the risk of any drug resistance due to target mutations [16]. Moreover, they are effective at low doses, minimizing the off-target toxicity associated with high-dose drugs [17].

## 4. PROTAC Disadvantages

**Figure 3:**
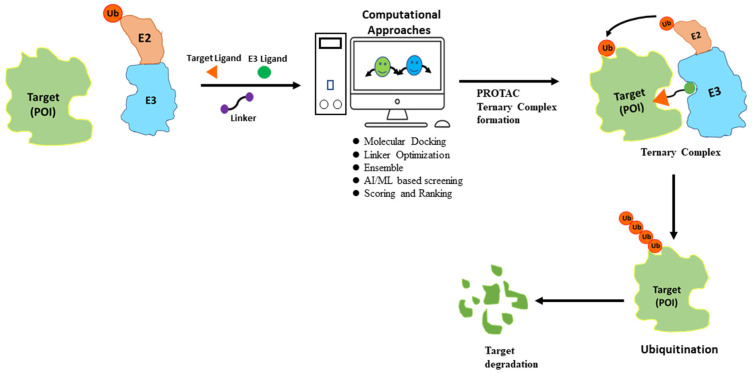
Flowchart for PROTAC discovery. Computational approaches can predict potential PROTAC molecules based on the target protein structure and small molecule ligand. The top predicted PROTACs can then be synthesized and experimentally evaluated for their ability to induce protein degradation. This iterative process of computational design and experimental evaluation can lead to the optimization of a PROTAC molecule with the desired potency, selectivity, and pharmacokinetic properties.

Despite its advantages, PROTAC also faces many challenges and limitations. One major drawback is the lack of knowledge of many E3 ligases, especially about tissue-specificity and expression patterns as well as their correlation to disease targets [18]. Another major drawback of PROTACs is the potential for off-target activity. This occurs when the small molecule ligands or the recruited E3 ligase interact with unintended proteins, leading to unwanted effects [19,20]. Therefore, careful consideration and screening of both ligands and E3 ligases are required to minimize off-target effects and enhance the specificity of PROTACs. The binding interactions between PROTACs, target proteins, and E3 ligases are currently based on empirical evidence and lack a strong theoretical foundation. Furthermore, the extent of target protein degradation’s impact on phenotypic responses and potential immune resistance in PROTAC treatment requires further investigation in future clinical trials. Despite facing certain limitations, the development of PROTACs has gained significant momentum in recent years owing to their immense potential for targeted protein degradation. To further improve the efficacy of PROTACs, scientists have turned to computational approaches for efficient design, utilizing techniques such as structure-based design and artificial intelligence methods (Figure 3).

## 5. E3 ligases in PROTAC

The degradation and turnover of proteins are crucial processes mediated by the Ubiquitin–Proteasome System. E3 ligases, as essential regulators of this system, play a pivotal role in protein degradation [21]. They are responsible for recognizing specific target proteins and facilitating their ubiquitination, marking them for subsequent degradation by the proteasome [22]. Given their essential role in protein ubiquitination, E3 ligases have become attractive targets for drug development. While the human genome contains more than 600 E3 ligases, only a limited number of them have been specifically targeted by PROTACs for the selective degradation of proteins of interest (POIs) [23,24]. Consequently, the exploration and characterization of novel E3 ligases hold the potential to broaden the scope of protein targets amenable to degradation through PROTAC technology. This expansion would pave the way for the development of more potent and targeted therapeutic interventions for a wide range of diseases. E3 ligases exhibit complex expression patterns that can vary widely depending on the tissue, tumor, and cellular compartment, highlighting the need for a greater understanding of the regulation and specificity of E3 ligases [25,26,27].

Small molecules that modulate E3 ligase activity hold great promise for therapeutic intervention in various diseases and the development of novel E3 ligases [28,29,30]. For example, Chan CH et al. [29] employed a structure-based virtual screening method to identify a potent Skp2-E3 ligase inhibitor. This inhibitor demonstrated significant in vivo anticancer activity and increased chemotherapeutic drug sensitivity, leading to a reduction in cancer cell survival. Similarly, Ohoka et al. [30] developed an AhR E3 ligase-based PROTAC for the degradation of CRABP1, whereas Li et al. [31] reported the development of a PROTAC based on DCAF15 E3 ligase that targets and causes degradation of BRD4. Additionally, Karki R et al. [32] introduced a novel approach where ligands of known E3 ligases are described by a simple and effective pharmacophore fingerprinting scheme known as Extended Reduced Graph (ErG). Each ErG bit forms the basis for a multi-class classification model where singular E3 ligase target proteins are used as labels. This is the first example of such a classification approach in the E3 ligase field. The resultant statistical model showed an accuracy of 93.8%, allowing it to assign the correct E3 ligase binder to previously known E3 ligases. Consequently, this approach enables the computational screening and filtering of large compound libraries by predicting the probability of each compound binding to different E3 ligases. These findings emphasize the promising potential of utilizing small molecules, or PROTACs, to target E3 ligases as an innovative therapeutic strategy for diseases that currently have limited treatment options.

Covalent bond-based approaches have become an attractive option for PROTAC development [33]. Such methods employ covalently reactive small molecules to target E3 ligases, in addition to traditional methods. In recent studies, Ward and colleagues [34] utilized covalent ligand screening methods to develop a PROTAC targeting BRD4 by recruiting RNF4 E34 ligase. In another study, Nomura and colleagues [35] employed a similar approach, targeting the FEM1B E3 ligase to degrade BRD4 and BCR-ABL. Pinch and colleagues [36] introduced a novel approach called COFFEE, which entails the covalent attachment of particular ligands to exposed cysteines on E3 ligases VHL and SPSB2. These modified ligases are then introduced into live cells through electroporation, resulting in the formation of functional E3 ubiquitin ligase complexes. Overall, covalent bond-based approaches provide a promising avenue for expanding the range of E3 ligases available for targeted protein degradation. Single-cell RNA sequencing (scRNA-seq) has become a highly effective technique for studying the gene expression profiles of individual cells [37]. It enables researchers to gain insights into the unique gene expression patterns exhibited by each cell [37,38]. By generating high-resolution expression profiles of E3 ligases in different cell types, tissues, or disease states, scRNA-seq enables the identification of cell-type-specific expression patterns that may have therapeutic implications [39]. Indeed, the information derived from scRNA-seq data can inform the development of selective protein degradation strategies that target E3 ligases expressed in specific cell types or disease contexts. Utilizing the capabilities of scRNA-seq, scientists can attain a more comprehensive understanding of the intricate regulatory networks involving E3 ligases and their involvement in disease development. This knowledge serves as a foundation for the exploration and development of innovative therapeutic approaches [40]. For example, scRNA-seq has been used to identify E3 ligases that are specifically expressed in cancer cells, such as TNBC and melanoma cells, which can be targeted for selective protein degradation using PROTAC [41,42].

The integration of genomic and proteomic data with scRNA-seq can provide a comprehensive understanding of E3 ligase expression in different cell types, tissues, or disease states. This, in turn, facilitates the development of strategies for selective protein degradation. These approaches can also identify E3 ligases that are not cell-type-specific but are critical for protein degradation in cancer cells. In addition to scRNA-seq, other high-throughput approaches, such as CRISPR screening, can also identify E3 ligases with specific proteins of interest [43,44]. Altogether, the integration of multiomics data can enable the prioritization and identification of E3 ligases for targeted protein degradation in cancer and other diseases. In one study, Medvar et al. [45] tackled the problem of identifying the most likely E3 ubiquitin ligase responsible for AQP2 ubiquitination. To accomplish this, they created a publicly available database of 377 human E3 ubiquitin ligases, primarily composed of HECT, RING, and U-box proteins. The construction of the database involved the application of a Bayesian technique, which utilized extensive proteome and transcriptome datasets to rank ubiquitin ligases probabilistically. This approach allowed the researchers to identify the most probable E3 ligase responsible for AQP2 ubiquitination. The database and methodology can be applied to other E3 ligase–target relationships, providing a valuable tool for understanding the complex biology of protein degradation. In another attempt, Park and colleagues [46] proposed a novel approach for predicting E3 ligase–target interactions using the CKSAAP approach, which considers pairs of amino acids that are k residues apart in the protein sequence. By analyzing the frequency of these pairs across a dataset of known E3–target relationships, the model can learn the patterns and features that are indicative of E3 ligase–target interactions. This approach enables the prediction of novel E3–target relationships based solely on the protein sequence without the need for labor-intensive experimental methods. The proposed approach was evaluated on an independent dataset using various standard quantitative measures, achieving an average accuracy of 70.63%. The results suggest that this framework provides a promising strategy for predicting E3–target interactions and has the potential to advance our understanding of cell biology and accelerate the development of new therapeutics. Palomba et al. [47] introduce ELIOT (E3 LIgase pocketOme navigaTor), an extensive platform designed for the development of novel PROTACs. ELIOT incorporates the pocketome information of E3 ligases and employs innovative 3D descriptors to accurately characterize the ligase pockets. It offers valuable features such as PROTAC-ability scores and similarity analyses, aiding in the design and optimization of PROTAC molecules. The platform also includes information on tissue specificity and degree of involvement in specific cancer types, enabling informed selection of E3 ligases for the design of PROTACs with improved specificity. In conclusion, these approaches have proven to be valuable for discovering E3 ligases and their ligands, leading to the development of clinical candidates and tools for further investigating E3 ligase biology.

## 6. Linker in PROTAC

The formation of a ternary complex between the E3 ligase and components of the protein of interest (POI) does not guarantee a functional outcome [48,49]. Therefore, we need a specific spatial configuration of these ternary complexes for degradation. The linker is a crucial aspect of PROTAC development, as it connects the two ligands for the target protein and E3 ubiquitin ligase [50]. Its role is pivotal in assessing the effectiveness, specificity, and pharmacokinetics of the resulting compounds. Therefore, linker optimization is necessary to ensure maximum binding affinity, efficient target protein degradation, and minimal off-target effects [51]. The major considerations for linker design in PROTACs are as follows: firstly, the length of the linker is crucial for maintaining the correct distance between the two moieties. Linkers that are too short can disrupt ternary complex formation and reduce PROTAC activity, while linkers that are too long can alter the molecule’s stability (Table 1). Hence, the ideal linker length needs to be assessed individually, and it can range from 12 to more than 20 carbon atoms depending on the specific case [51,52]. Secondly, linker flexibility is another important factor to consider. The linker should be flexible enough to allow the two ligands to adopt the correct orientation for efficient protein degradation. However, excessive flexibility can lead to decreased selectivity and increased off-target effects. Therefore, the linker’s degree of flexibility should be carefully balanced to achieve optimal activity and selectivity. Thirdly, the linker’s rigidity is essential for maintaining the correct orientation of the ligands and facilitating robust ternary complex formation. Incorporating chemical bonds or other structural elements that constrain the conformation of the linker can achieve this. The use of heterocyclic scaffolds such as piperazine and piperidine in the linker of PROTAC molecules resulted in the formation of a stable ternary complex and effective protein degradation [53,54]. Additionally, the incorporation of polar motifs such as pyridine and piperidine can modify the physicochemical properties of PROTACs, leading to improved aqueous solubility and cell permeability (Table 1). However, excessive rigidity can lead to decreased solubility, which can limit the delivery of PROTACs to the target protein. Fourthly, the linker’s cleavability is crucial for the efficient degradation of the target protein. Incorporating specific cleavable bonds or motifs into the linker can achieve this. However, it is essential to ensure that the linker is cleaved only by the intended target protein or E3 ubiquitin ligase and not by other cellular proteases. Finally, the linker’s solubility is also an important consideration for the efficient delivery of the PROTAC to the target protein. Efforts have been made to improve the solubility of PROTACs, such as incorporating the piperazine moiety to improve rigidity and solubility upon protonation [55].

Recent studies have focused on optimizing the linker length to achieve high selectivity and efficacy in PROTAC-mediated protein degradation (Table 2). Cyrus et al. [52] demonstrated that a 16 atom linker was optimal for degrading estrogen receptor (ER)-α, while Burslem et al. [56] found that increasing the linker size slightly could switch the degradation target from HER2 to EGFR. These studies underscore the importance of linker length and geometry in modulating degradation and selectivity in PROTAC-based protein degradation. A precise understanding of the optimal linker length for a given target protein can facilitate the design of effective and selective PROTACs, which can be of great therapeutic value in many diseases. Computational methods have revolutionized the field of PROTAC development. Protein–protein docking is a powerful approach that can be utilized to design the optimum linker distance between the binder and warhead in a structure-guided manner. Nowak et al. [57] utilized protein–protein docking techniques to optimize the linker distance between the binder and warhead moieties. Their findings suggested that shorter linkers enhance selectivity by reducing the number of potential conformations. Additionally, the attachment site of the linker has a notable influence on the overall metabolic stability and protein degradation characteristics of PROTACs. Bricelj et al. [58] demonstrated that the attachment site of the linker affects the aqueous stability and protein degradation properties of CRBN ligands. Computational methods such as protein–protein docking can aid in identifying the optimal site on the linker to attach the warhead without compromising critical interactions with the protein of interest. Bian et al. [59] utilized the docking pose of wogonin bound to CDK9 to identify the optimal position for attaching a linker without interfering with critical binding interactions. In summary, these computational approaches play a vital role in optimizing and addressing challenges associated with linkers. They are instrumental in advancing the field of targeted protein degradation and are expected to continue playing a pivotal role in future advancements. Imrie et al. [60] have recently proposed a machine learning-based approach, “DeLinker”, for the de novo design of linkers in PROTACs. The graph-based deep generative model utilizes 3D structural information to generate or replace the linker between two fragments in a molecule. The generative process can be controlled by specifying the desired linker length and the fragments to be linked. The study suggests that machine learning methods such as DeLinker can serve as an alternative to structure-based design for PROTAC development. This approach could be particularly useful in cases where structural information is not available, enabling the efficient generation of novel linkers with desired properties. Guo et al. [61] recently introduced a deep learning-based approach called Link–INVENT for fragment linking in PROTAC design. This method utilizes an adjustable scoring function that enables the specification of various multi-parameter optimization targets. Link–INVENT has been demonstrated to effectively explore optimal linker lengths within a defined range of physicochemical properties and control linker linearity and flexibility by selecting linear or ring-containing linkers and controlling the ratio of rotatable bonds in the linker. The promising results of this study highlight the potential of machine learning-based methods for the efficient design and optimization of PROTACs. Ting Kao et al. [62] introduced a deep neural network named “AIMLinker” to aid in the design and generation of drug-like PROTAC analogs. AIMLinker leverages the structural information obtained from related fragments to generate linkers capable of accommodating and incorporating these fragments. The network filters out non-druggable structures guided by protein–protein complexes to ensure the final molecules are drug-like. The generated molecules undergo molecular docking to test their robustness and feasibility based on various criteria. The results showed that the generated PROTAC molecules have similar structural information with superior binding affinity to binding pockets compared to existing CRBN-dBET6-BRD4 ternary complexes. The results of these findings indicate that AIMLinker possesses the capability to design compounds suitable for PROTAC molecules, offering enhanced chemical properties. Such advancements hold promise for facilitating the development of novel and highly effective targeted therapies. Youhai et al. [63] introduced a novel framework called DRlinker, which utilizes reinforcement learning to control fragment linking in compounds, ensuring desired attributes. The method proved effective in various tasks, including linker length control, log P optimization, and bioactivity prediction.

Neeser et al. [64] recently introduced ShapeLinker, an innovative approach for creating linkers from scratch. This method uses reinforcement learning with an autoregressive SMILES generator to perform fragment linking. It aims to optimize a composite score that considers essential physicochemical properties and introduces a novel point cloud alignment score based on attention mechanisms. ShapeLinker can generate linkers that meet both 2D and 3D criteria effectively, outperforming previous methods in generating novel linkers while assuming a specific target linker conformation. Most of these linker generation methods are limited to producing linkers in either 1D SMILES or 2D graph formats, neglecting the consideration of ternary structures. To overcome this limitation, Baiqing Li and colleagues [65] introduce a groundbreaking 3D linker generative model known as PROTAC–INVENT. This innovative model has the capacity not only to generate SMILES representations of PROTAC compounds but also to generate their putative 3D binding conformations, which are associated with the target protein and the E3 ligase. Furthermore, the model is trained using a reinforcement learning (RL) approach in conjunction with the generation of PROTAC structures to align with predefined 2D and 3D properties. Overall, designing optimal linkers is a complex process, requiring careful consideration of factors such as length, stability, and pharmacokinetic properties to maximize the potency and selectivity of the resulting molecules.

## 7. PROTAC Design Strategies

The use of PROTAC represents a promising therapeutic approach aimed at the selective degradation of disease-associated proteins [66,67,68]. However, the development of effective PROTACs is a complex and time-consuming process that requires extensive chemical testing and optimization. To address this challenge, computational approaches have been applied to aid in the various aspects of its development (Table 3). These approaches include molecular docking, molecular dynamic simulation, pharmacophores, and artificial techniques [69,70,71].

## 8. PROTAC Development Using Structure-Based Approaches

Molecular docking is a computational method used to predict the interaction between small molecules and target proteins [72]. It can be used to design PROTACs that have optimal binding affinity for both the target protein and the E3 ligase, which is necessary for the targeted degradation of the protein. The advancement in AlphaFold, which predicts protein structure and interactions, is considered one of the most promising tools for designing PROTAC constructs [73]. It can predict protein–protein complexes in the multimer variant, which can be helpful in correctly predicting the PROTAC-mediated PPI interfaces. Molecular dynamics simulation [74] is a widely employed technique for simulating the temporal behavior of atoms and molecules. This computational method is valuable for investigating the stability and dynamics of the ternary complex formed by the PROTAC, the target protein, and the E3 ligase, which facilitates a deeper understanding of its properties. Pharmacophores [75] refer to a collection of chemical and steric characteristics that are essential for a molecule to engage with a biological target. These features are crucial in understanding the molecular interactions and designing compounds with the desired activity against the target. This approach can be useful for predicting the optimal chemical properties of a PROTAC, such as the distance between the binding sites for the target protein and the E3 ligase, to maximize its degradation efficiency. Machine learning algorithms [76] offer the capability to train models for predicting the binding affinity between a PROTAC molecule, its target protein, and E3 ligase. By leveraging these algorithms, it becomes possible to reduce the number of experiments needed for optimization. Through the utilization of large-scale datasets, machine learning can uncover complex patterns and relationships, enabling the development of accurate predictive models. This approach facilitates the identification of PROTAC molecules with higher binding affinity, streamlines the drug discovery process, and contributes to the efficient design of targeted protein degradation therapies. Several successful examples of these computational approaches have been reported for the development of PROTACs with increased efficiency and selectivity. For example, Drummond et al. [77] have developed and validated four computational methods for generating ensembles of PROTAC-mediated ternary complexes using molecular docking and molecular dynamics simulations. These methods incorporate information about the target protein, E3 ligase, and candidate ligands and employ filters based on known crystal structures to ensure reasonable geometries and stability. The authors have successfully discriminated between the degradation behavior of wild-type and mutant proteins, as well as among different targets and PROTAC molecules, demonstrating the high accuracy of these computational methods. The techniques have the potential to guide the design and optimization of PROTACs a priori and predict their degradation behavior. This study provides a promising approach to accelerate the development of effective PROTACs. Zaidman et al. [78] have introduced PRosettaC, a novel method for modeling ternary complexes induced by a given PROTAC using structural information of the target and E3 ligase in complex with their binding ligand, a SMILES string of the PROTAC, and information about two anchor regions. The method selects the optimal ternary complex based on the Rosetta energy score and the clustering of complexes. Although PRosettaC has been tested only on CRBN/VHL ligases, it has the potential to assist in the design and optimization of novel and existing PROTACs. Bai et al. [79] developed a protocol for modeling PROTAC-mediated ternary complexes using Rosetta and OMEGA. The protocol involves screening the linker conformations to ensure their compatibility with the docked model, followed by refinement of the complete models. The study establishes a relationship between linker length and cellular activity and reveals that interactions with the E3 ligase can modulate target selectivity. These findings have the potential to support the development and refinement of PROTACs, leading to improved efficacy and selectivity for these molecules. In another attempt, Bai et al. [80] introduced a computational method based on the protein structure to predict the ubiquitination of target proteins triggered by cereblon-based PROTACs. Using Rosetta, they generated ternary complex ensembles and modeled multiple conformations of the CRL4A ligase complex. The approach predicted the ubiquitination efficiency by separating the ternary ensemble into productive and unproductive complexes. The authors validated their models and utilized their modeling workflow to forecast the efficiency and sites of ubiquitination for a range of cyclin-dependent kinases upon administration of TL12-186, a pan-kinase PROTAC. This work has the potential to accelerate the design and optimization of PROTACs by enabling the prediction of their degradation efficiency for a given target protein. Weng et al. [81] also described a computational protocol to predict PROTAC-mediated ternary complex structures by combining local docking by FRODOCK and structure refinement by RosettaDock, along with several filters and re-scoring algorithms. The authors provided evidence to support the superior performance of the FRODOCK-based protocol compared to other existing methods in accurately modeling the near-native structures of ternary complexes, starting from the unbound structures. Tu et al. [82] developed specific PROTAC degraders of EZH2, which outperformed traditional inhibitors in inhibiting lymphomas in vitro and in vivo. They used the EZH2 inhibitor EPZ6438 to design two series of PROTAC-based EZH2 degraders that recruit different E3 ligase systems, VHL or CRBN. Through molecular docking analysis protocols integrated into MOE and in vitro experiments, they identified compounds YM181 and YM281 as the most effective EZH2 degraders targeting the VHL E3 ligase. Accurate prediction of the ternary pose is crucial for designing structure-based PROTACs when the actual structure of the ternary complex is unavailable. However, it is worth noting that not every generated ternary complex is robust for ubiquitination. Liao et al. [83] proposed HAPOD (Heating-Accelerated Pose Departure) to rank and score hypothetical ternary complex poses in the absence of a known ternary co-crystal structure. The method uses protein–protein docking and MD simulation to generate potential structures and provides an assessment for structure-based PROTAC design. The study highlights how to advance PROTAC development in the absence of crystal structures. Gaining insights into the free energy of binding to a target necessitates an understanding of the conformational behavior of small molecules in a free solution. This is particularly relevant for proteolysis-targeting chimeras (PROTACs) due to their inherent flexibility, length, and the requirement to form a ternary complex. Weerakoon et al. [84] conducted MD simulations and utilized NMR data to characterize the conformational space of two PROTAC molecules, MZ1 and dBET6. Their findings revealed that conformations featuring a hydrophobic contact between the two warheads exhibited slightly favorable tendencies. Wenqing et al. [85] proposed a protocol to optimize initial ternary complexes generated by Rosetta using MD simulation and MM/GBSA. The authors also investigated the “hook effect” of a specific PROTAC and proposed a cooperativity factor, α. Their work provides insights into the binding and dynamics of PROTACs, as well as the impact of flexible linkers on ternary complexes. In a recent study by Mai et al. [86], a novel computational framework was introduced to model the cooperativity between PROTAC-E3 binding and PROTAC-target binding using a coarse-grained (CG) approach. Their CG approach effectively captures the essential aspects of cooperativity, including the identification of optimal intermediate linker lengths resulting from configurational entropy.

In a study by Yokoo et al. [87], a previous PROTAC molecule was optimized using docking simulations. The resulting PROTAC, H-PGDS-7 (6), demonstrated potent and selective degradation activity by inhibiting prostaglandin D2 production in KU812 cells. It also exhibited superior inhibition of inflammatory cytokines in a Duchenne muscular dystrophy model compared to a potent H-PGDS inhibitor, TFC-007. This research underscores the potential of docking simulations for the design and optimization of PROTAC molecules. In another recent study, Rao et al. [88] introduced a method called BOTCP (Bayesian Optimization for Ternary Complex Prediction) to accelerate and refine the prediction of PROTAC ternary complexes. The approach involved simulated annealing MD simulations combined with molecular mechanics generalized Born surface area (MMGBSA) scoring to assign high rankings, even to small clusters with experimentally determined structures. However, despite observed improvements, consistent attainment of top-ranking near-native models was still challenging using this method.

## 9. PROTAC Development Using Machine Learning

Zheng et al. [89] proposed a deep generative model that uses deep reinforcement learning for the efficient design of PROTACs in low-resource settings. The model optimizes compounds with desirable pharmacokinetics for a given target protein. The research identified six potential PROTAC candidates, and subsequent validation was performed through cell-based assays and Western blot analysis on three of these candidates. One candidate showed favorable pharmacokinetics in mice. This approach can facilitate rational PROTAC design and optimization using deep learning and molecular simulations and has potential applications in drug discovery. DeepPROTACs [90] is another deep neural network model designed to predict the efficacy of PROTACs in degrading the target protein of interest (POI). The model incorporates the structures of both the POI and the E3 ligase and consists of separate neural network modules for different parts of the POI-PROTAC-E3 ligase complex. The model achieved an average accuracy rate of 77.95% and an AUROC of 0.8470 on the test set. Validation using PROTACs recruiting VHL to degrade estrogen receptor (ER) showed a prediction accuracy of 68.75% for 11 out of 16 PROTACs. While this study demonstrates promising results, the authors acknowledge the need to address certain limitations, such as considering the distance between solvent-exposed lysines on the POI and the E3-ligase complex, which may play a critical role in degradation efficacy. Nori et al. [91] explored the use of AI, specifically graph-based generative models and reinforcement learning, for designing effective PROTACs with improved chemical properties and reduced off-target effects. The generative model suggested molecules with substructures similar to those found in known degraders. Through fine-tuning, the predicted activity against the target protein of interest (POI) increased from 50% to over 80% while maintaining high chemical validity. This study highlights the potential of AI for optimizing PROTACs for targeted protein degradation. Zhang et al. [92] developed a machine-learning model called MAPD, which uses intrinsic protein features to predict protein degradability. The model accurately predicts the degradability of kinases by TPD compounds, achieving an AUROC of 0.775 and an AUPRC of 0.759. The model can likely be applied to non-kinase proteins as well. Through statistical analysis, five features were identified as significant predictors, with ubiquitination potential being the most predictive. Structural modeling revealed the importance of E2-accessible ubiquitination sites for kinase degradability rather than general lysine residues. MAPD predictions were extended to the entire proteome, identifying 964 disease-causing proteins, including those encoded by 278 cancer genes, that could be targeted for TPD drug development. This study showcases the potential of machine learning in predicting protein degradability and identifying disease-causing proteins for drug development. These findings collectively demonstrate the potential of computational approaches, including deep learning, generative models, and machine learning, in PROTAC design, ternary complex prediction, and protein degradability prediction. They provide insights into the development of novel PROTAC-based therapeutics and highlight the role of computational methods in advancing targeted protein degradation strategies. Anticipating cell permeability holds significant importance in streamlining the development of low-permeable PROTACs, thus conserving resources for synthesis and testing. Recently, Poongavanam et al. [93] developed predictive binary classification models for PROTAC cell permeability. These models were applied to a diverse collection of cereblon (CRBN) and von Hippel−Lindau (VHL) PROTACs, revealing insights into their potential and limitations. For the VHL PROTAC dataset, both k-nearest neighbor and random forest models exhibited superior performance, accurately predicting blinded test sets with over 80% precision. Retraining models with combined original training and blinded test sets yielded consistent results for a separate blinded VHL set. Conversely, models for CRBN PROTACs faced challenges, primarily due to the imbalanced nature of the CRBN datasets. Although all descriptors contributed to the models, size and lipophilicity emerged as the most influential. In summary, properly trained machine learning models have the potential to serve as effective filters in the PROTAC design process. While progress has been made in generative models for PROTACs, current methods mainly focus on 2D structures, overlooking the fit within the PROTAC ternary complex binding site. To address this limitation, ref. [94] conducted a benchmark study to evaluate computational tools for PROTAC design. Three different methods (ICM, MOE, and PRosettaC) were evaluated for predicting ternary complex structures and screening PROTAC libraries. While some accurate predictions of protein-protein interfaces were observed, efficient PROTAC virtual screening remains unclear, with active PROTACs not consistently ranking high. Crystal structures may not represent the only relevant ternary complexes, as active PROTAC-induced conformations differ. More experimental data on active and inactive PROTACs is needed for method development and evaluation. Filtering based on ubiquitination zones did not improve predictions, but specific active PROTAC conformations show promise, warranting further exploration.

**Table 3:** Models for predicting the PROTACs.

Model	Method	Description	Ref
Zheng S. et al.	Deep reinforcement learning in combination with machine learning-based classifiers and MD simulations.	Applying this method to the bromodomain-containing protein 4 target protein. Model optimizes compounds with desirable pharmacokinetics for a given target protein.	[89]
DeepPROTACs	Graph Convolutional Networks.	Dataset from PROTAC–DB was used to perform the modeling. Can predict the ability of given PROTACs to induce the degradation of a specific POI by recruiting a specific E3 ligase.	[90]
Nori D. et al.	Graph-based generative models and reinforcement learning.	Effective PROTACs with improved chemical properties and reduced off-target effects. Model-suggested molecules have substructures similar to those found in known degraders.	[91]
MAPD	Naïve Bayes, KNN, LR LiblineaR, SVM_Linear_, SVM_Radial_, and Random Forest.	Uses intrinsic protein features to predict protein degradability. Predict kinases that are degradable by TPD compounds.	[92]
Poongavanam V. et al.	Random Forest, Decision Tree, Support Vector Machine, and Kappa Nearest Neighbor.	Binary classification models. Models were constructed using training sets of 113 CRBN and 115 VHL PROTACs. Predict the permeability of CRBN and VHL PROTACs.	[93]

## 10. Conclusions and Future Direction

Over the past decades, PROTACs have made significant advancements in both academics and industry. They offer a promising and powerful approach to target disease proteins that are currently considered undruggable by small molecules or are difficult to drug, thus overcoming a significant hurdle in drug discovery. However, to fully exploit the potential of PROTACs, further investigation into E3 ligase biology, including tissue-specificity, expression patterns, and disease targets, is necessary. The next generation of PROTACs is expected to exploit novel ligands to target more E3 ligases and offer disease-specific degradation therapies. E3 ligases in PROTAC development offer immense potential but are accompanied by noteworthy limitations. One primary challenge lies in the limited diversity of E3 ligases available for PROTAC design, which can restrict the range of targetable proteins. In addition, tissue-specific protein degradation remains another hurdle, as many diseases require precise localization. Minimizing off-target effects or toxicity is another important factor that needs to be considered and requires meticulous fine-tuning. As discussed, the length of the linker used in PROTACs is a key factor influencing how effectively they can break down target proteins. It is like the bridge between two important components: the molecule that targets the protein we want to degrade and the one that attaches to the E3 ligase. The chemical properties of these linkers also matter a great deal. They must maintain stability within the natural conditions of our body and demonstrate proficiency while moving into the cells. These linkers should have a strong binding affinity to their respective molecules to ensure that the proteins can bind effectively, which is necessary for the degradation process. Thus, proper linker length, chemical properties, binding strength, and spatial arrangement are essential for developing effective PROTAC therapies.

Computational technologies, like deep learning and structure-based approaches, provide significant opportunities to address the challenges in PROTAC development. Deep learning algorithms can analyze large datasets, find the best linkers, forecast binding affinities, and help improve ternary complex structures. These technologies can speed up PROTAC design by narrowing down which ones are likely to work well and be safe (Table 3). Structure-based approaches, on the other hand, leverage molecular modeling and simulation techniques to predict the spatial arrangement of proteins within ternary complexes. By simulating the interactions at the atomic level, these methods provide insights into the stability and feasibility of PROTAC-induced protein degradation. Moreover, they allow for the virtual screening of candidate PROTACs against various E3 ligases, expanding the toolbox of available ligands. However, achieving a consensus approach that seamlessly integrates the strengths of both deep learning and structure-based techniques is crucial (Table 3). By combining predictive power with structural insights, researchers can make informed decisions about PROTAC design, linker selection, and target engagement. As more crystal structures of ternary complexes become available, these computational approaches will become even more accurate, providing a roadmap for the future of PROTAC development. In summary, computational technologies, including deep learning and structure-based approaches, hold the potential to revolutionize PROTAC research. Their ability to accelerate candidate screening, optimize ternary complex structures, and guide ligand selection offers a promising direction toward developing safer and more effective drugs through PROTACs. We believe that continued research in this direction will be instrumental in harnessing the full potential of these PROTAC-based therapeutics. Advancements in computational models for protein–protein interactions and ternary complex formation are poised to revolutionize structure-based PROTAC design.

## Figures and Tables

**Table 1:** Key features of linkers for PROTAC development.

Features	Description
**Length**	The length of the linker is a critical parameter as it determines the spatial distance between the ligands targeting the protein of interest (POI) and the E3 ligase. Optimal linker length is essential for facilitating the proper orientation and binding of the ligands to their respective targets. Varies, typically 12–20 atoms.
**Flexibility**	The flexibility of the linker influences the conformational freedom of the ligands and their ability to engage with the POI and E3 ligase. Consideration of the target proteins and their relative orientations is significant in determining the required flexibility of the linker.
**Chemical ** **Composition**	Composed of various chemical moieties, such as polyethylene glycol (PEG), Alkyne, Triazole, Piperazine, Piperidine, or other organic structures. Can affect the stability, solubility, and pharmacokinetics of the PROTAC.
**Cleavability**	Cleavable linkers are designed to be sensitive to cellular conditions, leading to the release of ligands and subsequent degradation of the POI. Non-cleavable linkers remain intact throughout the process, allowing for continuous ternary complex formation.
**Cell Permeability**	Designing linkers with optimum cell permeability is crucial for ensuring the efficient delivery of PROTAC into target cells.
**Hydrophilicity/** **Hydrophobicity**	The solubility and cell membrane penetration of the PROTAC molecule can be affected by the linker’s hydrophilic or hydrophobic properties.
**Specificity**	To prevent off-target effects, linker design should take the specificity of interaction with the desired POI and E3 ligase into account.
**In vivo Stability**	Consideration of the stability of the linker in the physiological environment is essential for the successful application of PROTACs in vivo.
**Structural ** **Diversity**	Structural diversity enables the customization of different targets.

**Table 2:** Models for linker development and optimization.

Model	Method	Description	Ref
DeLinker	Graph-based deep generative.	Leverages three-dimensional structural data to generate the linker connecting two fragments. The generation process can be regulated by specifying the linker length and specifying the fragments to be connected.	[60]
Link–INVENT	Recurrent Neural Network (RNN) and Reinforcement Learning.	Generate favorable linkers connecting two molecules. Flexible scoring function.	[61]
AIMLinker	Graph Neural Network (GNN).	Incorporate structural 3D information. Can filter the non-druggable structures.	[62]
DRlinker	Deep Reinforcement Learning.	Regulate the linking of fragments to create compounds with specific attributes. The method has demonstrated effectiveness across numerous tasks, including controlling linker length and log P and optimizing the predicted bioactivity of compounds.	[63]
ShapeLinker	Reinforcement Learning.	Conducts fragment linking through reinforcement learning using an autoregressive SMILES generator. The method successfully produces linkers that meet both pertinent 2D and 3D criteria.	[64]
PROTAC–INVENT	Reinforcement Learning.	Can generate 3D putative binding conformation coupled with the target protein and the E3 ligase.	[65]

## Data Availability

Data sharing is not applicable.

## References

[1] Burslem G.M., Crews C.M. (2020). Proteolysis-Targeting Chimeras as Therapeutics and Tools for Biological Discovery. Cell.

[2] Zou Y., Ma D., Wang Y. (2019). The PROTAC technology in drug development. Cell Biochem. Funct..

[3] Békés M., Langley D.R., Crews C.M. (2022). PROTAC targeted protein degraders: The past is prologue. Nat. Rev. Drug. Discov..

[4] Bond M.J., Crews C.M. (2021). Proteolysis targeting chimeras (PROTACs) come of age: Entering the third decade of targeted protein degradation. RSC Chem. Biol..

[5] Zhao L., Zhao J., Zhong K., Tong A., Jia D. (2022). Targeted protein degradation: Mechanisms, strategies and application. Sig. Transduct. Target. Ther..

[6] Pei H., Peng Y., Zhao Q., Chen Y. (2019). Small molecule PROTACs: An emerging technology for targeted therapy in drug discovery. RSC Adv..

[7] Burke M.R., Smith A.R., Zheng G. (2022). Overcoming Cancer Drug Resistance Utilizing PROTAC Technology. Front. Cell Dev. Biol..

[8] He M., Cao C., Ni Z., Liu Y., Song P., Hao S., He Y., Sun X., Rao Y. (2022). PROTACs: Great opportunities for academia and industry (an update from 2020 to 2021). Sig. Transduct. Target. Ther..

[9] Hu Z., Crews C.M. (2022). Recent Developments in PROTAC-Mediated Protein Degradation: From Bench to Clinic. ChemBioChem.

[10] Qi S.M., Dong J., Xu Z.Y., Cheng X.D., Zhang W.D., Qin J.J. (2021). (PROTAC: An Effective Targeted Protein Degradation Strategy for Cancer Therapy. Front. Pharmacol..

[11] Sakamoto K.M., Kim K.B., Kumagai A., Mercurio F., Crews C.M., Deshaies R.J. (2001). PROTACS: Chimeric molecules that target proteins to the Skp1-Cullin-F box complex for ubiquitination and degradation. Proc. Natl. Acad. Sci. USA.

[12] Xie H., Liu J., Alem Glison D.M., Fleming J.B. (2021). The clinical advances of proteolysis targeting chimeras in oncology. Explor. Target. Antitumor. Ther..

[13] Kelm J.M., Pandey D.S., Malin E., Kansou H., Arora S., Kumar R., Gavande N.S. (2023). PROTAC’ing oncoproteins: Targeted protein degradation for cancer therapy. Mol. Cancer.

[14] Weng G., Shen C., Cao D., Gao J., Dong X., He Q., Yang B., Li D., Wu J., Hou T. (2021). PROTAC-DB: An online database of PROTACs. Nucleic Acids Res..

[15] Weng G., Cai X., Cao D., Du H., Shen C., Deng Y., He Q., Yang B., Li D., Hou T. (2023). PROTAC-DB 2.0: An updated database of PROTACs. Nucleic Acids Res..

[16] Kim H., Park J., Kim J.M. (2022). Targeted Protein Degradation to Overcome Resistance in Cancer Therapies: PROTAC and N-Degron Pathway. Biomedicines.

[17] Liu Z., Hu M., Yang Y., Du C., Zhou H., Liu C., Chen Y., Fan L., Ma H., Gong Y. (2022). An overview of PROTACs: A promising drug discovery paradigm. Mol. Biomed..

[18] Martín P.A., Xiao X. (2021). PROTACs to address the challenges facing small molecule inhibitors. Eur. J. Med. Chem..

[19] Kannt A., Đikić I. (2021). Expanding the arsenal of E3 ubiquitin ligases for proximity-induced protein degradation. Cell Chem. Biol..

[20] Liu Y., Yang J., Wang T., Luo M., Chen Y., Chen C., Ronai Z., Zhou Y., Ruppin E., Han L. (2023). Expanding PROTACtable genome universe of E3 ligases. Nat. Commun..

[21] Ishida T., Ciulli A. (2021). E3 Ligase Ligands for PROTACs: How They Were Found and How to Discover New Ones. SLAS Discov..

[22] Belcher B.P., Ward C.C., Nomura D.K. (2023). Ligandability of E3 Ligases for Targeted Protein Degradation Applications. Biochemistry..

[23] Sampson C., Wang Q., Otkur W., Zhao H., Lu Y., Liu X., Piao H.L. (2023). The roles of E3 ubiquitin ligases in cancer progression and targeted therapy. Clin. Transl. Med..

[24] Yang Q., Zhao J., Chen D., Wang Y. (2021). E3 ubiquitin ligases: Styles, structures and functions. Mol. Biomed..

[25] Humphreys L.M., Smith P., Chen Z., Fouad S., D’Angiolella V. (2021). The role of E3 ubiquitin ligases in the development and progression of glioblastoma. Cell Death Differ..

[26] Diehl C.J., Ciulli A. (2022). Discovery of small molecule ligands for the von Hippel-Lindau (VHL) E3 ligase and their use as inhibitors and PROTAC degraders. Chem. Soc. Rev..

[27] Michaelides I.N., Collie G.W. (2023). E3 Ligases Meet Their Match: Fragment-Based Approaches to Discover New E3 Ligands and to Unravel E3 Biology. J. Med. Chem..

[28] Wang G., Chan C.H., Gao Y., Lin H.K. (2012). Novel roles of Skp2 E3 ligase in cellular senescence, cancer progression, and metastasis. Chin. J. Cancer.

[29] Chan C.H., Morrow J.K., Li C.F., Gao Y., Jin G., Moten A., Stagg L.J., Ladbury J.E., Cai Z., Xu D. (2013). Pharmacological inactivation of Skp2 SCF ubiquitin ligase restricts cancer stem cell traits and cancer progression. Cell.

[30] Ohoka N., Tsuji G., Shoda T., Fujisato T., Kurihara M., Demizu Y., Naito M. (2019). Development of Small Molecule Chimeras That Recruit AhR E3 Ligase to Target Proteins. ACS Chem. Biol..

[31] Li L., Mi D., Pei H., Duan Q., Wang X., Zhou W., Jin J., Li D., Liu M., Chen Y. (2020). In vivo target protein degradation induced by PROTACs based on E3 ligase DCAF15. Signal. Transduct. Target. Ther..

[32] Karki R., Gadiya Y., Gribbon P., Zaliani A. (2023). Pharmacophore-Based Machine Learning Model To Predict Ligand Selectivity for E3 Ligase Binders. ACS Omega.

[33] Collins K.H., Winter G.E., Bernardes G.L. (2021). The role of reversible and irreversible covalent chemistry in targeted protein. Cell Chem. Biol..

[34] Ward C.C., Kleinman J.I., Brittain S.M., Lee P.S., Chung C.Y.S., Kim K., Petri Y., Thomas J.R., Tallarico J.A., McKenna J.M. (2019). Covalent Ligand Screening Uncovers a RNF4 E3 Ligase Recruiter for Targeted Protein Degradation Applications. ACS Chem. Biol..

[35] Henning N.J., Manford A.G., Spradlin J.N., Brittain S.M. (2022). Discovery of a Covalent FEM1B Recruiter for Targeted Protein Degradation Applications. J. Am. Chem. Soc..

[36] Pinch B.J., Buckley D.L., Gleim S., Brittain S.M., Tandeske L., D’Alessandro P.L., Hauseman Z.J., Lipps J., Xu L., Harvey E.P. (2022). A strategy to assess the cellular activity of E3 ligase components against neo-substrates using electrophilic probes. Cell Chem. Biol..

[37] Haque A., Engel J., Teichmann S.A., Lönnberg T.A. (2017). A practical guide to single-cell RNA-sequencing for biomedical research and clinical applications. Genome Med..

[38] Jovic D., Liang X., Zeng H., Lin L., Xu F., Luo Y. (2022). Single-cell RNA sequencing technologies and applications: A brief overview. Clin. Transl. Med..

[39] Hoch M., Rauthe J., Cesnulevicius K., Schultz M., Lescheid D., Wolkenhauer O., Chiurchiù V., Gupta S. (2023). Cell-Type-Specific Gene Regulatory Networks of Pro-Inflammatory and Pro-Resolving Lipid Mediator Biosynthesis in the Immune System. Int. J. Mol. Sci..

[40] Ding S., Chen X., Shen K. (2020). Single-cell RNA sequencing in breast cancer: Understanding tumor heterogeneity and paving roads to individualized therapy. Cancer Commun..

[41] He Y., Khan S., Huo Z., Lv D., Zhang X., Liu X., Yuan Y., Hromas R., Xu M., Zheng G. (2020). Proteolysis targeting chimeras (PROTACs) are emerging therapeutics for hematologic malignancies. J. Hematol. Oncol..

[42] Kaneko M., Iwase I., Yamasaki Y., Takai T., Wu Y., Kanemoto S., Matsuhisa K., Asada R., Okuma Y., Watanabe T. (2016). Genome-wide identification and gene expression profiling of ubiquitin ligases for endoplasmic reticulum protein degradation. Sci. Rep..

[43] Lin K., Shen S.H., Lu F., Zheng P., Wu S., Liao J., Jiang X., Zeng G., Wei D. (2022). CRISPR screening of E3 ubiquitin ligases reveals Ring Finger Protein 185 as a novel tumor suppressor in glioblastoma repressed by promoter hypermethylation and miR-587. J. Transl. Med..

[44] Bock C., Datlinger P., Chardon F., Coelho M.A., Dong M.B., Lawson K.A., Lu T., Maroc L., Norman T.M., Song B. (2022). High-content CRISPR screening. Nat. Rev. Methods Primers.

[45] Medvar B., Raghuram V., Pisitkun T., Sarka A., Knepper M.A. (2016). Comprehensive database of human E3 ubiquitin ligases: Application to aquaporin-2 regulation. Physiol. Genom..

[46] Park S., Khan S., Wahab A. (2020). E3-targetpred: Prediction of e3-target proteins using deep latent space encoding. arXiv.

[47] Palomba T., Baroni M., Cross S., Cruciani G., Siragusa L. (2023). ELIOT: A platform to navigate the E3 pocketome and aid the design of new PROTACs. Chem. Biol. Drug Des..

[48] Hanzl A., Casement R., Imrichova H., Hughes S.J., Barone E., Testa A., Bauer S., Wright J., Brand M., Ciulli A. (2023). Functional E3 ligase hotspots and resistance mechanisms to small-molecule degraders. Nat. Chem. Biol..

[49] Li K., Crews C.M. (2022). PROTACs: Past, present and future. Chem. Soc. Rev..

[50] Bemis T.A., La Clair J.J., Burkart M.D. (2021). Unraveling the Role of Linker Design in Proteolysis Targeting Chimeras. J. Med. Chem..

[51] Cecchini C., Pannilunghi S., Tardy S., Scapozza L. (2021). From Conception to Development: Investigating PROTACs Features for Improved Cell Permeability and Successful Protein Degradation. Front. Chem..

[52] Cyrus K., Wehenkel M., Choi E.Y., Han H.J., Lee H., Swanson H., Kim K.B. (2011). Impact of linker length on the activity of PROTACs. Mol. Biosyst..

[53] Farnaby W., Koegl M., Roy M.J., Whitworth C., Diers E., Trainor N., Zollman D., Steurer S., Karolyi-Oezguer J., Riedmueller C. (2019). BAF complex vulnerabilities in cancer demonstrated via structure-based PROTAC design. Nat. Chem. Biol.

[54] Han X., Wang C., Qin C., Xiang W., Fernandez-Salas E., Yang C.Y., Wang M., Zhao L., Xu T., Chinnaswamy K. (2019). Discovery of ARD-69 as a highly potent proteolysis targeting chimera (PROTAC) degrader of Androgen Receptor (AR) for the treatment of prostate cancer. J. Med. Chem..

[55] Desantis J., Mammoli A., Eleuteri M., Coletti A., Croci F., Macchiarulo A., Goracci L. (2022). PROTACs bearing piperazine-containing linkers: What effect on their protonation state?. RSC. Adv..

[56] Burslem G.M., Smith B.E., Lai A.C., Jaime-Figueroa S., McQuaid D.C., Bondeson D.P., Toure M., Dong H., Qian Y., Wang J. (2018). The advantages of targeted protein degradation over inhibition: An RTK case study. Cell Chem. Biol..

[57] Nowak R.P., DeAngelo S.L., Buckley D., He Z., Donovan K.A., An J., Safaee N., Jedrychowski M.P., Ponthier C.M., Ishoey M. (2018). Plasticity in binding confers selectivity in ligand-induced protein degradation. Nat. Chem. Biol..

[58] Bricelj A., Steinebach C., Kuchta R., Gütschow M., Sosič I. (2021). E3 Ligase Ligands in Successful PROTACs: An Overview of Syntheses and Linker Attachment Points. Front. Chem..

[59] Bian J., Ren J., Li Y., Wang J., Xu X., Feng Y., Tang H., Wang Y., Li Z. (2018). Discovery of Wogonin-based PROTACs against CDK9 and capable of achieving antitumor activity. Bioorg. Chem..

[60] Imrie F., Bradley A.R., van der Schaar M., Deane C.M. (2020). Deep Generative Models for 3D Linker Design. J. Chem. Inf. Model..

[61] Guo J., Knuth F., Margreitter C., Janet J.P., Papadopoulos K., Engkvist O., Patronov A. (2023). Link-INVENT: Generative Linker Design with Reinforcement Learning. Digit. Discov..

[62] Kao T.C., Lin T.C., Chou L.C., Lin C.C. (2023). Fragment Linker Prediction Using Deep Encoder-Decoder Network for PROTAC Drug Design. J. Chem. Inf. Model..

[63] Tan Y., Dai L., Huang W., Guo Y., Zheng S. (2022). DRlinker: Deep Reinforcement Learning for Optimization in Fragment Linking Design. J. Chem. Inf. Model..

[64] Neeser R.M., Akdel M., Kovtun D., Naef L. (2023). Reinforcement Learning-Driven Linker Design via Fast Attention-based Point Cloud Alignment. arXiv.

[65] Li B., Ran T., Chen H. (2023). 3D Based Generative PROTAC Linker Design with Reinforcement Learning. Brief. Bioinform..

[66] Smith B.E., Wang S.L., Figueroa J.S., Harbin A., Wang J., Hamman B.D., Crews C.M. (2019). Differential PROTAC substrate specificity dictated by orientation of recruited E3 ligase. Nat. Commun..

[67] Bondeson D.P., Smith B.E., Burslem G.M., Buhimschi A.D., Hines J., Jaime-Figueroa S., Wang J., Hamman B.D., Ishchenko A., Crews C.M. (2018). Lessons in PROTAC design from selective degradation with a promiscuous warhead. Cell Chem. Biol..

[68] Němec V., Schwalm M.P., Müller S., Knapp S. (2022). PROTAC degraders as chemical probes for studying target biology and target validation. Chem. Soc. Rev..

[69] Samarasinghe K.T.G., Crews C.M. (2021). Targeted protein degradation: A promise for undruggable proteins. Cell Chem. Biol..

[70] Salama A.K.A.A., Trkulja M.V., Casanova E., Uras I.Z. (2022). Targeted Protein Degradation: Clinical Advances in the Field of Oncology. Int. J. Mol. Sci..

[71] He S., Dong G., Cheng J., Wu Y., Sheng C. (2022). Strategies for designing proteolysis targeting chimaeras (PROTACs). Med. Res. Rev..

[72] Vakser I.A. (2014). Protein-protein docking: From interaction to interactome. Biophys. J..

[73] Pereira G.P., Jiménez-García B., Pellarin R., Launay G., Wu S., Martin J., Souza P.C.T. (2023). Rational Prediction of PROTAC-Compatible Protein-Protein Interfaces by Molecular Docking. J. Chem. Inf. Model..

[74] Zhang L., Buck M. (2013). Molecular simulations of a dynamic protein complex: Role of salt-bridges and polar interactions in configurational transitions. Biophys. J..

[75] Hu B., Lill M.A. (2013). Exploring the potential of protein-based pharmacophore models in ligand pose prediction and ranking. J. Chem. Inf. Model..

[76] Guo Z., Yamaguchi R. (2022). Machine learning methods for protein-protein binding affinity prediction in protein design. Front. Bioinform..

[77] Drummond M.L., Williams C.I. (2019). In Silico Modeling of PROTAC-Mediated Ternary Complexes: Validation and Application. J. Chem. Inf. Model..

[78] Zaidman D., Prilusky J., London N. (2020). PRosettaC: Rosetta Based Modeling of PROTAC Mediated Ternary Complexes. J. Chem. Inf. Model..

[79] Bai N., Miller S.A., Andrianov G.V., Yates M., Kirubakaran P., Karanicolas J. (2021). Rationalizing PROTAC-Mediated Ternary Complex Formation Using Rosetta. J. Chem. Inf. Model..

[80] Bai N., Riching K.M., Makaju A., Wu H., Acker T.M., Ou S.C., Zhang Y., Shen X., Bulloch D.N., Rui H. (2020). Modeling the CRL4A ligase complex to predict target protein ubiquitination induced by cereblon-recruiting PROTACs. J. Biol. Chem..

[81] Weng G., Li D., Kang Y., Hou T. (2021). Integrative Modeling of PROTAC-Mediated Ternary Complexes. J. Med. Chem..

[82] Tu Y., Sun Y., Qiao S., Luo Y., Liu P., Jiang Z.X., Hu Y., Wang Z., Huang P., Wen S. (2021). Design, Synthesis, and Evaluation of VHL-Based EZH2 Degraders to Enhance Therapeutic Activity against Lymphoma. J. Med. Chem..

[83] Liao J., Nie X., Unarta I.C., Ericksen S.S., Tang W. (2022). In Silico Modeling and Scoring of PROTAC-Mediated Ternary Complex Poses. J. Med. Chem..

[84] Weerakoon D., Carbajo R.J., De Maria L., Tyrchan C., Zhao H. (2022). Impact of PROTAC Linker Plasticity on the Solution Conformations and Dissociation of the Ternary Complex. J. Chem. Inf. Model..

[85] Li W., Zhang J., Guo L., Wang Q. (2022). Importance of Three-Body Problems and Protein-Protein Interactions in Proteolysis-Targeting Chimera Modeling: Insights from Molecular Dynamics Simulations. J. Chem. Inf. Model..

[86] Mai H., Zimmer M.H., Miller T.F. (2023). Exploring PROTAC Cooperativity with Coarse-Grained Alchemical Methods. J. Phys. Chem..

[87] Yokoo H., Shibata N., Endo A., Ito T., Yanase Y. (2021). Discovery of a Highly Potent and Selective Degrader Targeting Hematopoietic Prostaglandin D Synthase via In Silico Design. J. Med. Chem..

[88] Rao A., Tunjic T.M., Brunsteiner M., Michael M., Hosein F., Noah W. (2022). Bayesian Optimization for Ternary Complex Prediction (BOTCP). Artif. Intell. Life Sci..

[89] Zheng S., Tan Y., Wang Z., Li C., Zhang Z., Sang X., Chen H., Yang Y. (2022). Accelerated rational PROTAC design via deep learning and molecular simulations. Nat. Mach. Intell..

[90] Li F., Hu Q., Zhang X., Sun R., Liu Z., Wu S., Tian S., Ma X., Dai Z., Yang X. (2022). DeepPROTACs is a deep learning-based targeted degradation predictor for PROTACs. Nat. Commun..

[91] Nori D., Coley C.W., Mercado R. (2022). De novo PROTAC design using graph-based deep generative models. arXiv.

[92] Zhang W., Roy Burman S.S., Chen J., Donovan K.A., Cao Y., Shu C., Zhang B., Zeng Z., Gu S., Zhang Y. (2022). Machine Learning Modeling of Protein-intrinsic Features Predicts Tractability of Targeted Protein Degradation. Genom. Proteom. Bioinform..

[93] Poongavanam V., Kölling F., Giese A., Göller A.H., Lehmann L., Meibom D., Kihlberg J. (2023). Predictive Modeling of PROTAC Cell Permeability with Machine Learning. ACS Omega.

[94] Rovers E., Schapira M. (2023). Benchmarking of PROTAC docking and virtual screening tools. bioRxiv.

